# Mental Health Response Vehicles in Wales: A Pilot Initiative

**DOI:** 10.1192/bjo.2025.10156

**Published:** 2025-06-20

**Authors:** Mark Jones

**Affiliations:** Joint Commissioning Committee, Cardiff, United Kingdom

## Abstract

**Aims:** Emergency NHS services are under considerable pressures from patient demand and ineffectual care and social pathways. This is especially felt within the mental health services where demand has grown from long periods of austerity and the Covid pandemic. To reduce demand on both ambulances and emergency departments Welsh ambulance implemented the roll out of mental health practitioners within its 999 call centres which has been very successful, however, the successful closure/treatment rate is less than half the callers. Over half of all mental health 999 callers require face to face intervention therefore ambulances within Wales need to implement mental health response vehicles in order to achieve this.

**Methods:** To test this further between January and March this year a pilot was conducted in Aneurin Bevan University Health Board over a 9-week period operating Friday to Sunday 1 pm to 12 am.

**Results:** The pilot achieved very positive outcomes in staff satisfaction and perceived patient satisfaction, along with 74% see, treat and close at scene rate, 7% conveyance to mental health facilities and 19% Emergency department conveyance rate.

**Conclusion:** The project has been a huge success and demonstrates how working in partnerships across organisations can achieve significant success through overcoming barriers as and when they arise. Mental health patients can face long waits and psychological hardships whilst they wait for treatments. MHRV can provide not only rapid responsive assessments but also provide a therapeutic intervention to those patients, thereby improving quality of care to mental health patients and releasing further ED and ambulance resource to the general public.

Finally, a national rollout of this way of working will require a significant investment, both financially and staffing; obtaining suitably qualified, experienced and skilled staff will represent a significant burden upon Welsh health boards or English Trusts. English ambulance trusts have already faced issues with recruiting staff into their services, which may be the same in WAST or if successful will mean that health boards will lose staff from within their organisations placing an additional burden upon their services (in 2023 the National Commissioning and Collaborative Unit reported that there were circa 500 mental health nurse vacancies across Wales).

The authors propose that a partnership approach is required with the Welsh NHS executive, HEIW, Welsh universities and health boards to explore training options and create a national mental health education package for Wales. WAST will be able to train and equip its existing staff with mental health knowledge and skills to treat patients utilising the ambulance service.

